# The impact of dormitory atmosphere on academic performance in medical university: a cross-sectional study

**DOI:** 10.3389/fpsyg.2025.1677658

**Published:** 2025-10-27

**Authors:** Ying Xu, Rujing Xu, Junjie Wu, Yuehan Wen, Nuo Xu, Yiwen Gao, Zixin Liang, Jianzhong Wang, Jie Hu, Xiaqiu Wu, Binyan Wang, Jing Fang

**Affiliations:** ^1^School of Public Health, Zhejiang Chinese Medical University, Hangzhou, Zhejiang, China; ^2^Department of Student Affairs, Zhejiang Chinese Medical University, Hangzhou, Zhejiang, China; ^3^Academic Affairs Office, Zhejiang Chinese Medical University, Hangzhou, Zhejiang, China

**Keywords:** dormitory hygiene, dormitory academic atmosphere, dormitory interpersonal atmosphere, academic performance, higher education

## Abstract

**Introduction:**

Academic performance (AP) serves as a comprehensive measure of students’ academic proficiency and learning status, while dormitory atmosphere plays a significant role in shaping students’ daily lives. This study investigated the impact of dormitory atmosphere on the AP of college students.

**Methods:**

A questionnaire-based survey was conducted at Zhejiang Chinese Medical University, incorporating the grade point average (GPA) of each participant as part of the research data. The study included 601 participants.

**Results:**

The study found that dormitory hygiene and dormitory interpersonal atmosphere were significantly correlated with AP (OR = 0.54, 95% CI [0.36, 0.83], *p* = 0.004; OR = 1.60, 95% CI [1.10, 2.31], *p* = 0.014), whereas the dormitory academic atmosphere within the dormitory showed no significant association (OR = 0.91, 95% CI [0.62, 1.33], *p* = 0.624). Subgroup analysis revealed that dormitory hygiene (OR = 2.86, 95% CI [1.70, 4.82], *p* < 0.001) and dormitory interpersonal atmosphere (OR = 0.41, 95% CI [0.25, 0.66], *p* < 0.001) significantly influenced AP among female students, whereas no significant effects were observed in male students.

**Discussion:**

These findings provide valuable insights into factors influencing AP and offer guidance for creating a supportive dormitory environment for college students.

## 1 Introduction

Academic performance (AP), which serves as a comprehensive indicator of students’ learning level and learning status (Wang et al., 2022), has highly predictive effect on their future career development (Peng et al., 2021). Students with an outstanding AP may receive scholarships, job offers, and even be recommended to pursue a graduate degree at prestige universities. Conversely, students with poor AP may be difficult in finding desirable employment opportunities and may not even be successful in graduating (Zayed et al., 2022). Therefore, it is crucial to investigate the factors that impact their AP and intervene accordingly and timely so that their AP could be ideal.

Dormitories, in these years, have become more than traditional residential spaces and particularly conducive to people’s informal and non-institutionalized learning activities (Allaste et al., 2022; Thomsen, 2007). A recent study found that College students are even going through a shift from traditional classroom that offers formal education to this informal and flexible learning environments (Valtonen et al., 2021). In China, almost all universities adopt a full-time boarding system for undergraduates. Dormitories are one of the most frequently used spaces throughout students’ college years (He and Zeng, 2025), making this residential environment of university students critical yet understudied determinant of AP (Thompson et al., 1993).

Dormitory atmosphere is the overall cognition of dormitory environment of college students. It is a relatively lasting psychological cognition widely shared by dormitory members, and a representation of dormitory characteristics, events and operation process. It usually includes the material environment and non-material environment of dormitory (Yancui, 2007; Jin, 2002). Most studies suggested dormitory environment is positively correlated with students’ better AP (Wayne et al., 2013; Desai et al., 2022; Sharma et al., 2024; Berhanu and Sewagegn, 2024). Bad dormitory environment can destroy students’ sleep, lead to mental disorder, thereby harming their AP (Li et al., 2022; Darvishi et al., 2025). Additionally, as one of the imperative social elements in universities, the interpersonal atmosphere of dormitory is also associated with students’ academic achievements (Khursheed and Baig, 2014). By analyzing longitudinal data from 5,272 undergraduate students at a university in China, where roommates were randomly assigned and lived together for a long period of time, a previous study found that the similarity of roommates’ AP in the actual data was significantly higher than that expected from a random assignment model (i.e., “the roommate zero model”) (Cao et al., 2024). This assimilation effect increased with the amount of time roommates lived together, suggesting the great influence of the non-material environment of dormitory on students’ AP.

Although dormitory affects students’ AP in multiple ways, some evidence predicts gender differences in the impact of dorm environments on student achievement. Functional imaging studies reveal that women exhibit stronger neurochemical responses to social stressors (e.g., interpersonal conflicts), which may heighten sensitivity to dormitory dynamics (Pasion et al., 2023). For instance, Magolda and Astin found that social interactions in residential settings have a significant impact on AP, and that females may be more susceptible to environmental influences (Astin, 1993). Pascarella and Terenzini noted that social support in residential settings may have a greater impact on AP for women than for men (Pascarella and Terenzin, 2005). Although these studies explored the differences in many aspects between male and female, there are still relatively few studies available exploring the differential impact of dormitory on male and female AP (Pascarella and Terenzin, 2005).

As microcosms of social, academic, and personal development, dormitories intersect with three key dimensions—dormitory hygiene, dormitory academic atmosphere, and dormitory interpersonal atmosphere—each of which may uniquely and synergistically shape students’ educational outcomes (Sharma et al., 2022; Ismail et al., 2020; Collie et al., 2016; Martin, 2014; Rania et al., 2014). While existing research had explored some effects of dormitory (de Araujo and Murray, 2010; Manson-Dioso and Iglesia, 2021), the interplay of these three dimensions, alongside gender-specific dynamics, remained poorly understood.

Therefore, this study aimed to investigate the interaction of college dormitory hygiene, academic atmosphere interpersonal atmosphere in China and their differential influence on male and female undergraduates’ AP through a cross-sectional study. We hoped this study could have some implication for a positive and long-term significance for future education policies.

## 2 Methods

### 2.1 Study design

This was an explorative and cross-sectional study that was conducted at Zhejiang Chinese Medicine University (ZCMU), which is located in the Yangtze River Delta region of China. Upon enrollment, the students were usually housed in the dormitories on campus provided by the university. There were four to six students lived in one room, and the students were randomly allocated without consideration of other factors such as student origin and ethnicity. A self-reported questionnaire (Wen, 2006; Wu, 2020; Hu, 2006; Guo, 2016; Ren, 2021) was conducted between May and August of 2023 in the dormitories on the campus and their study situation and dorm atmosphere were surveyed. Grade point average (GPA) was provided by ZCMU’s Academic Affairs Office. This study was approved by the ethical committee of ZCMU (No. 20230427-2).

### 2.2 Subjects

In this study, full-time undergraduates enrolled at ZCMU between 2018 and 2022 were recruited. The inclusion criteria for participant selection were: (1) reside long-term in the dormitory provided by the university; (2) normal participation in courses and examinations; and (3) demonstration of sufficient cognitive ability to provide reliable responses to the questionnaire.

Eligible students were interviewed, recruited and informed on ZCMU campus, with the informed consent forms obtained from all enrollees and the questionnaires completed electronically via QR code or on paper.

### 2.3 Measurements

#### 2.3.1 Dormitory atmosphere as the exposure

Based on “Impact in the roommate effect on the college students’ AP questionnaire” (Hu, 2018), an “Undergraduate dormitory atmosphere questionnaire” for data collection was designed, which contained a total of 24 questions and was divided into 3 parts: socio-demographic characteristics, personal ability and habit, dormitory atmosphere (dormitory hygiene, dormitory academic atmosphere, and dormitory interpersonal atmosphere) questionnaire. Indexes including the KMO coefficient and Cronbach’s alpha are calculated to evaluate the validity and reliability of the questionnaire (KMO = 0.737, Cronbach’s alpha = 0.327). The KMO coefficient ranges from 0 to 1. The closer the coefficient is to 1, the higher the structural validity of the questionnaire. Kaiser proposed that a KMO value of 0.5 is barely acceptable, 0.7 to 0.8 is considered good, and above 0.9 is excellent (Kaiser and Rice, 1974; Bernard et al., 2020; Tun, 2025; Habib et al., 2025). The KMO value in this study was 0.737, indicating that the factors derived from the analysis possess high reliability and confirming that the sample size was adequate. The detailed questionnaire and the scoring criteria can be seen in Supplementary Table S2. Additionally, we conduct reliability tests for each dimension separately to see the reliability for each dimension, which was shown in Supplementary Table S3.

##### 2.3.1.1 Dormitory hygiene score

This study investigated the frequency of individual cleaning, the frequency of group cleaning, and the results of actual school dormitory hygiene inspection. Dormitories were rated as failing, fair, good, or excellent based on inspection results. Individual cleaning frequency, group cleaning frequency, and actual school dormitory hygiene inspection results were assigned points. The cumulative score of the last three indicators was the indicator of personal dormitory hygiene. The total indicator of dormitory hygiene score was obtained by adding the individual dormitory hygiene indicators of students in the same dormitory.

##### 2.3.1.2 Dormitory academic atmosphere score

This study measured the dormitory academic atmosphere from four aspects: frequency of learning communication behavior, frequency of learning information sharing, individual learning engagement, and additional learning engagement under peer influence. The dormitory academic atmosphere score is obtained by adding up the individual academic atmosphere index of students in the same dormitory.

##### 2.3.1.3 Dormitory interpersonal atmosphere score

This study investigated the harmonious degree of dormitory interpersonal communication from three aspects: communication, collective consciousness, and conflict. Negative indicators such as conflict are given a negative score, while positive indicators such as communication and collective consciousness are given a positive score. Personal interpersonal communication index is obtained by the accumulation of scores, and the Dormitory interpersonal atmosphere score of students in the same dormitory is added to get the dormitory interpersonal atmosphere index.

#### 2.3.2 Academic performance as the main outcomes

Academic performance (AP) was assessed through a combination of GPA, competition results and research papers. Grade point average (GPA) is a system used in high schools and colleges to measure a student’s performance and academic achievement (Ultimate Lexicon, 2025). For the purpose of adjusting for restriction of range, current GPA was calculated for every student in this study (Westrick, 2017). Due to the variety and gold content of university students’ discipline competitions, only the awards of Zhejiang provincial level and above are included in the discipline competitions, and the cumulative statistics of the number of awards are carried out. The score of scientific research papers is calculated by adding up the number of published papers, because considering the difficulty of publishing papers for undergraduates, the quality of papers is not subdivided. In the actual data processing, it is found that less than 1% of the students have won prizes in competitions and published papers. These two variables are excluded, and only GPA is considered as the criterion for judging AP. Participants’ GPA was obtained through the academic affairs system in Zhejiang Chinese Medical University and those with GPAs in the top 30% of their grades in the same major are judged to have high AP according to the “Zhejiang Chinese Medicine University Undergraduate Scholarship Evaluation Measures.”

### 2.4 Other variables as confounders

Variables represent baseline characteristics of the participants, and identified as potential confounding factors that could exert an influence on the outcome based on previous studies (Afolabi, 2019; Jobbehdar Nourafkan et al., 2020) or discussions conducted in the research team, were considered in this study and grouped into two categories as follows: (1) socio-demographic characteristics, including sex (male, female), maternal education (junior high school or below, high school, bachelor, master or above), paternal education (junior high school or below, high school, bachelor, master or above), monthly expense (<$200, $200–$600, >$600), learning atmosphere in the family (poor, fair, good) (defined by how many books you have in home); and (2) personal ability and habit, including student leadership experience (no, yes), honors (no, yes), early to bed (seldom, often, daily) (defined by the number of days in a week when the bedtime point is around 23:30 or before), early to rise (seldom, often, daily) (defined by the number of days in a week when the wake-up time is around 8:00 or before), study time (less, average, more).

### 2.5 Statistical analysis

Enrollees’ basic characteristics are quantitatively summarized: medians and interquartile ranges (IQR) for non-normally distributed continuous data, and frequencies and proportions for categorical data. Kolmogorov–Smirnov test was used to assess the normality of continuous variable distributions in the dataset. All continuous did not satisfy the assumption of normality, variable dormitory hygiene, dormitory academic atmosphere, and dormitory interpersonal atmosphere were treated with median binary classification.

Binomial logistic regression models, a model used to study the association between binary dependent variable and any independent variable, were applied to assess the association between dormitory atmosphere and AP using SPSS 25.0. AP, which was assessed by GPA, was set as the dependent variable. Covariant, such as student leadership experience, honors, maternal and paternal education, monthly expenses, family learning atmosphere, sleep habits (early to bed and early to rise), and study time, were chosen as the independent variable. To explore the independent and interactive effects of dormitory hygiene, academic atmosphere and interpersonal atmosphere on AP, we have set four model incorporating additional independent variable. Model 1–3 additionally incorporated dormitory hygiene, dormitory academic atmosphere and dormitory interpersonal atmosphere as the independent variable, respectively. Model 4 included all three dormitory-related factors (dormitory hygiene, dormitory academic atmosphere, and dormitory interpersonal atmosphere). And to explore the moderating effect of sex, we calculated the strength of the association between dormitory atmosphere and AP in both the male and female subgroups.

Prior to conducting regression analysis with the adjustments for the selected covariates, we assessed multicollinearity among the independent variables by calculating variance inflation factors (VIFs). No multicollinearity was found (Supplementary Table S1). A two-sided *p*-value <0.05 was deemed statistically significant. Statistical analyses were conducted using R version 4.3.2 and SPSS 25.

## 3 Results

### 3.1 Sample characteristics

A total of 601 participants were enrolled, and Table 1 presents their baseline characteristics. The median values for Dormitory Hygiene, dormitory academic atmosphere, dormitory interpersonal atmosphere and GPA were 2.67 (IQR: 1.00), 1.95 (IQR: 0.40), 2.80 (IQR: 0.40), and 3.05 (IQR: 0.74), respectively. The majority of participants were female (58.60%), had student leadership experience (69.20%), and had received honors (58.80%). In addition, the majority reported maternal education at junior high school or below (46.10%), paternal education at junior high school or below (43.80%), and monthly expenses ranging between $200 and $600 (94.00%). Furthermore, a significant proportion perceived their family learning atmosphere as poor (48.60%), reported a habit of going to bed early (59.90%) and waking up early (79.50%), and considered their study time to occupy an average proportion of their extracurricular life (88.70%). Furthermore, the study indicates that 65.60% of the students are categorized as low achievers in terms of their AP.

**Table 1:** Enrollee characteristics.

Characteristics	*N* (%) (*N* = 601)	Low achievers (%) (*N* = 394)	High achievers (%) (*N* = 207)	*P*
Sex				<0.001
Male	249 (41.40)	191 (48.48)	58 (28.02)	
Female	352 (58.60)	203 (51.52)	149 (71.98)	
Student leadership experience				0.008
No	416 (69.20)	287 (72.84)	129 (62.32)	
Yes	185 (30.80)	107 (27.16)	78 (37.68)	
Honors				<0.001
No	353 (58.80)	266 (67.51)	88 (42.51)	
Yes	247 (41.20)	128 (32.49)	119 (57.49)	
Maternal education				0.487
Junior high school or below	277 (46.10)	189 (47.97)	88 (42.51)	
High school	211 (35.10)	131 (33.25)	80 (38.65)	
Bachelor	95 (15.80)	61 (15.48)	34 (16.43)	
Master or above	18 (3.00)	13 (3.30)	5 (2.42)	
Paternal education				0.216
Junior high school or below	263 (43.80)	175 (44.42)	88 (42.51)	
High school	210 (34.90)	140 (35.53)	70 (33.82)	
Bachelor	105 (17.50)	61 (15.48)	44 (21.26)	
Master or above	23 (3.80)	18 (4.57)	5 (2.42)	
Monthly expense				0.154
<$200	25 (4.20)	18 (4.57)	7 (3.38)	
$200–$600	565 (94.00)	366 (92.89)	199 (96.14)	
>$600	11 (1.80)	10 (2.54)	1 (0.48)	
Learning atmosphere in the family				0.623
Poor	292 (48.60)	189 (47.97)	103 (49.76)	
Fair	269 (44.80)	176 (44.67)	93 (44.93)	
Good	40 (6.70)	29 (7.36)	11 (5.31)	
Early to bed				0.912
Seldom	175 (29.10)	117 (29.70)	58 (28.02)	
Often	360 (59.90)	234 (59.39)	126 (60.87)	
Daily	66 (11.00)	43 (10.91)	23 (11.11)	
Early to rise				0.877
Seldom	27 (4.50)	18 (4.57)	9 (4.35)	
Often	478 (79.50)	311 (78.93)	167 (80.68)	
Daily	96 (16.00)	65 (16.50)	31 (14.98)	
Study time				0.019
Less	19 (3.20)	13 (3.30)	6 (2.90)	
Average	533 (88.70)	340 (86.29)	193 (93.24)	
More	49 (8.20)	41 (10.41)	8 (3.86)	
Dormitory hygiene score, median (IQR)	2.67 (1.00)	2.67 (1.00)	2.33 (1.06)	<0.001
Dormitory academic atmosphere score, median (IQR)	1.95 (0.40)	2.00 (0.50)	1.90 (0.35)	0.004
Dormitory interpersonal atmosphere score, median (IQR)	2.80 (0.40)	2.80 (0.40)	2.84 (0.33)	0.414
Dormitory hygiene				<0.001
Low	255 (42.40)	138 (35.03)	117 (56.52)	
High	346 (57.60)	256 (64.97)	90 (43.48)	
Dormitory academic atmosphere				0.085
Low	299 (49.80)	186 (47.21)	113 (54.59)	
High	302 (50.20)	208 (52.79)	94 (45.41)	
Dormitory interpersonal atmosphere				0.065
Low	275 (45.80)	191 (48.48)	84 (40.58)	
High	326 (54.20)	203 (51.52)	123 (59.42)	
GPA, median (IQR)	3.05 (0.74)	2.85 (0.75)	3.34 (30.54)	<0.001
AP				
Low achievers	394 (65.6)	/	/	
High achievers	207 (34.4)	/	/	

GPA, grade point average; AP, academic performance.

### 3.2 Regression analysis between dormitory atmosphere and AP

Table 2 presents the results of logistic regression analyses examining the association between dormitory atmosphere and AP, adjusted for sex, student leadership experience, academic honors, parental education levels, monthly expenses, family learning atmosphere, sleep patterns, and study time, revealing that dormitory hygiene was negatively associated with AP (Model 1: OR = 0.54, 95% CI [0.36, 0.83], *p =* 0.004), dormitory academic atmosphere showed no statistically significant relationship with AP (Model 2: OR = 0.91, 95% CI [0.62, 1.33], *p =* 0.624), and dormitory interpersonal atmosphere was positively correlated with AP (Model 3: OR = 1.60, 95% CI [1.10, 2.31], *p* = 0.014), while in the combined analysis (Model 4), dormitory hygiene (OR = 0.56, 95% CI [0.37, 0.85], *p =* 0.006) and dormitory interpersonal atmosphere (OR = 1.57, 95% CI [1.08, 2.29], *p =* 0.018) remained significantly associated with AP, and dormitory academic atmosphere continued to exhibit no significant correlation.

**Table 2:** Regression analysis between dormitory atmosphere and AP.

Variable	Model 1			Model 2			Model 3			Model 4		
	OR	95%CI	*P*	OR	95%CI	*P*	OR	95%CI	*P*	OR	95%CI	*P*
Sex												
Male	Ref			Ref			Ref			Ref		
Female	1.55	0.98, 2.43	0.061	2.05	1.35, 3.11	0.001	2.22	1.48, 3.31	<0.001	1.58	0.99, 2.54	0.057
Student leadership experience												
No	Ref			Ref			Ref			Ref		
Yes	1.17	0.78, 1.75	0.454	1.17	0.78, 1.75	0.445	1.14	0.76, 1.70	0.537	1.12	0.75, 1.69	0.579
Honors												
No	Ref			Ref			Ref			Ref		
Yes	2.41	1.63, 3.57	<0.001	2.44	1.65, 3.60	<0.001	2.43	1.65, 3.60	<0.001	2.44	1.65, 3.63	<0.001
Maternal education												
Junior high school or below	Ref			Ref			Ref			Ref		
High school	1.57	0.98, 2.52	0.060	1.57	0.98, 2.52	0.058	1.63	1.01, 2.61	0.043	1.61	1.00, 2.59	0.051
Bachelor	1.15	0.56, 2.38	0.696	1.25	0.61, 2.56	0.542	1.29	0.63, 2.65	0.486	1.19	0.58, 2.46	0.631
Master or above	1.28	0.34, 4.87	0.718	1.36	0.36, 5.15	0.652	1.43	0.37, 5.48	0.602	1.39	0.36, 5.35	0.630
Paternal education												
Junior high school or below	Ref			Ref			Ref			Ref		
High school	0.81	0.51, 1.29	0.373	0.83	0.52, 1.32	0.426	0.82	0.52, 1.32	0.417	0.81	0.50, 1.29	0.368
Bachelor	1.30	0.66, 2.57	0.456	1.27	0.64, 2.51	0.491	1.27	0.64, 2.52	0.496	1.31	0.66, 2.61	0.445
Master or above	0.61	0.17, 2.24	0.456	0.61	0.17, 2.22	0.455	0.52	0.14, 1.92	0.329	0.51	0.14, 1.88	0.310
Monthly expense												
<$200	Ref			Ref			Ref			Ref		
$200–$600	1.08	0.39, 2.97	0.882	1.12	0.41, 3.09	0.827	1.10	0.40, 3.03	0.861	1.10	0.39, 3.04	0.861
>$600	0.34	0.03, 3.71	0.374	0.37	0.03, 4.03	0.412	0.39	0.04, 4.29	0.439	0.35	0.03, 3.88	0.390
Learning atmosphere in the family												
Poor	Ref			Ref			Ref			Ref		
Fair	0.84	0.56, 1.26	0.396	0.80	0.54, 1.20	0.289	0.79	0.53, 1.19	0.254	0.82	0.55, 1.24	0.343
Good	0.65	0.28, 1.52	0.319	0.63	0.27, 1.46	0.278	0.60	0.25, 1.41	0.240	0.62	0.26, 1.46	0.275
Early to bed												
Seldom	Ref			Ref			Ref			Ref		
Often	1.11	0.72, 1.69	0.642	1.13	0.74, 1.72	0.585	1.14	0.75, 1.74	0.553	1.13	0.74, 1.73	0.581
Daily	1.61	0.81, 3.20	0.172	1.51	0.76, 2.99	0.240	1.58	0.79, 3.16	0.194	1.66	0.83, 3.31	0.154
Early to rise												
Seldom	Ref			Ref			Ref			Ref		
Often	0.63	0.24, 1.59	0.331	0.61	0.24, 1.54	0.297	0.56	0.22, 1.41	0.220	0.56	0.22, 1.43	0.224
Daily	0.51	0.18, 1.42	0.197	0.51	0.18, 1.42	0.197	0.48	0.17, 1.35	0.164	0.47	0.17, 1.34	0.160
Study time												
Less	Ref			Ref			Ref			Ref		
Average	1.11	0.34, 3.57	0.867	1.14	0.35, 3.72	0.822	1.18	0.36, 3.88	0.790	1.12	0.34, 3.63	0.856
More	0.38	0.10, 1.53	0.175	0.38	0.09, 1.53	0.172	0.39	0.10, 1.60	0.193	0.39	0.10, 1.57	0.184
Dormitory hygiene												
Low	Ref									Ref		
High	0.54	0.36, 0.83	0.004							0.56	0.37, 0.85	0.006
Dormitory academic atmosphere												
Low				Ref						Ref		
High				0.91	0.62, 1.33	0.624				0.91	0.62, 1.35	0.653
Dormitory interpersonal atmosphere												
Low							Ref			Ref		
High							1.60	1.10, 2.31	0.014	1.57	1.08, 2.29	0.018

Model 1: adjusted for sex, student leadership experience, honors, maternal education, paternal education, monthly expense, learning atmosphere in the family, early to bed, early to rise, study time, dormitory hygiene. *R*^2^ = 0.17.

Model 2: adjusted for sex, student leadership experience, honors, maternal education, paternal education, monthly expense, learning atmosphere in the family, early to bed, early to rise, study time, dormitory academic atmosphere. *R*^2^ = 0.16.

Model 3: adjusted for sex, student leadership experience, honors, maternal education, paternal education, monthly expense, learning atmosphere in the family, early to bed, early to rise, study time, dormitory interpersonal atmosphere. *R*^2^ = 0.17.

Model 4: adjusted for sex, student leadership experience, honors, maternal education, paternal education, monthly expense, learning atmosphere in the family, early to bed, early to rise, study time, dormitory hygiene, dormitory academic atmosphere, dormitory interpersonal atmosphere. *R*^2^ = 0.18.

### 3.3 The association between dormitory atmosphere and AP stratified by sex

Table 3 presents gender-stratified analysis of the association between dormitory atmosphere and AP. In the male cohort, no significant associations were observed between dormitory atmosphere (dormitory hygiene, dormitory academic atmosphere and dormitory interpersonal atmosphere) and AP. In contrast, among females, dormitory hygiene showed a significant positive correlation with AP (Model 1: OR = 2.95, 95% CI [1.78, 4.88], *p <* 0.001), while dormitory interpersonal atmosphere exhibited a significant negative correlation (Model 3: OR = 0.38, 95% CI [0.24, 0.61], *p <* 0.001). In the combined analysis (Model 4), no significant association with AP was observed among males, while among females, dormitory hygiene remained positively correlated (OR = 2.86, 95% CI [1.70, 4.82], *p <* 0.001), and dormitory interpersonal atmosphere showed a persistent negative correlation (OR = 0.41, 95% CI [0.25, 0.66], *p <* 0.001).

**Table 3:** Association between dormitory atmosphere and AP stratified by sex.

Model	Male (*N* = 249)			Female (*N* = 352)		
	OR	95%CI	*P*	OR	95%CI	*P*
Model 1 – Dormitory hygiene	0.30	0.08, 1.07	0.063	2.95	1.78, 4.88	<0.001
Model 2 – Academic atmosphere	0.71	0.34, 1.47	0.356	1.36	0.85, 2.19	0.201
Model 3 – Dormitory interpersonal atmosphere.	1.60	0.84, 3.08	0.155	0.38	0.24, 0.61	<0.001
Model 4 – Dormitory hygiene	0.29	0.08, 1.08	0.065	2.86	1.70, 4.82	<0.001
Model 4 – Dormitory academic atmosphere	0.83	0.38, 1.80	0.634	1.53	0.93, 2.53	0.094
Model 4 – Dormitory interpersonal atmosphere	1.81	0.93, 3.56	0.083	0.41	0.25, 0.66	<0.001

Model 1: adjusted for sex, student leadership experience, honors, maternal education, paternal education, monthly expense, learning atmosphere in the family, early to bed, early to rise, study time. *R*^2^ = 0.17 *R*^2^ = 0.19 for male and female, respectively.

Model 2: adjusted for sex, student leadership experience, honors, maternal education, paternal education, monthly expense, learning atmosphere in the family, early to bed, early to rise, study time. *R*^2^ = 0.15 *R*^2^ = 0.13 for male and female, respectively.

Model 3: adjusted for sex, student leadership experience, honors, maternal education, paternal education, monthly expense, learning atmosphere in the family, early to bed, early to rise, study time. *R*^2^ = 0.16 *R*^2^ = 0.18 for male and female, respectively.

Model 4: adjusted for sex, student leadership experience, honors, maternal education, paternal education, monthly expense, learning atmosphere in the family, early to bed, early to rise, study time, dormitory hygiene, dormitory academic atmosphere, dormitory interpersonal atmosphere. *R*^2^ = 0.19, *R*^2^ = 0.24 for male and female, respectively.

## 4 Discussion

This study found that the dormitory atmosphere is significantly correlated with students’ AP. Specifically, dormitory hygiene demonstrated a negative correlation with AP, whereas interpersonal relationships within the dormitory exhibited a positive correlation. However, no significant association was observed between the academic atmosphere in dormitories and students’ AP.

Contrary to studies emphasizing the benefits of hygiene for students’ AP (Ramli et al., 2021; Rafiq and Kamran, 2022), our results revealed a negative correlation between dormitory hygiene and AP. This may be attributed to the Simpson’s Paradox (Hernán et al., 2011). It’s a common statistical phenomenon that indicates simply summarizing grouped data may lead to misleading conclusions. Gender, as an important confounding factor between dormitory atmosphere and AP, distorts the causal relationship between the two in the combined analysis and fails to reflect the true relationship. In our study, the women’s dormitory atmosphere scores and AP were significantly higher than the men’s., which caused a distortion and generated a weak positive correlation trend in the pooled regression. The merged ‘positive correlation’ may only reflect the overall trend of females having higher AP and higher dormitory atmosphere, completely masking the true relationship between AP and dormitory atmosphere within each group. Consequently, we conducted subgroup analysis on males and females in this study. We found a positive correlation between dormitory hygiene and AP in women, but no correlation in men. This may be due to the fact that women’s hygiene norms are stricter than men’s (Eriksson et al., 2022), which leads to the stronger influence of dormitory hygiene on women. Women are likely to be more sensitive to dormitory hygiene, which makes poor dormitory hygiene more likely to affect their AP. Male students’ academic achievement was more strongly tied to extracurricular engagement (e.g., sports teams, part-time jobs) than to residential environments, suggesting that off-campus identity-building activities buffer institutional influences (Crosnoe and Muller, 2014). Additionally, dormitory hygiene scores were based on administrators’ subjective observations (e.g., bed tidiness) rather than objective indicators (e.g., microbiological testing). In ZCMU, the proportion of female managers is significantly higher than that of male managers. Due to the interpersonal relation and the different living habits of males and females, female administrators tend to give lower scores to male dormitory than female dormitory (Joshi et al., 2015). This subjectivity may result in scores that are decoupled from real hygiene conditions and do not reflect actual health gains, thereby masking positive associations or reinforcing false negative correlations.

The lack of association between academic atmosphere and GPA contradicts earlier work highlighting peer-driven study norms (Rania et al., 2014; Ismail et al., 2020; Sacerdote, 2001; Booij et al., 2017; Zimmerman, 2003). This discrepancy likely reflects evolving learning behaviors in the digital age. The proliferation of smartphone uses and the availability of a variety of educational apps has prompted a departure from traditional learning methods (Hattie, 2023). Also, Demir and Akpınar found that the students appreciated mobile learning as an approach that may significantly increase their motivation (Demir and Akpınar, 2018). Furthermore, self-regulated learners (Zimmerman and Risemberg, 1997; Theobald, 2021) may buffer environmental distractions, rendering peer study habits less impactful.

The findings of this study suggest a significant positive correlation between interpersonal atmosphere in dormitories and students’ AP in merged analysis. This aligns with prior research emphasizing the critical role of interpersonal atmosphere in shaping educational outcomes (Cao et al., 2024; Khan et al., 2023). However, some striking results were found in the subgroup analysis, which found an inverse correlation between dorm room interpersonal climate and academic achievement among women, while no correlation was found among men. The paradox can also be explained by the Simpson’s Paradox (Hernán et al., 2011) caused by genders. And the reason for the difference between males and females can be related to that neurobehavioral and sociocultural factors may amplify gender disparities in dormitory impacts. Women exhibit heightened HPA-axis activation to social stressors (e.g., interpersonal conflicts), amplifying dormitory impacts (Brydges et al., 2020). Additionally, a previous study also found that women are more likely to maintain harmonious dormitory relationships, and they may spend more time on non-academic social activities (such as emotional exchanges, group activities), thereby crowding out study time and harming their AP.

Our study refined the dormitory atmosphere into three core dimensions: dormitory hygiene, dormitory academic atmosphere, and dormitory interpersonal atmosphere, explored their independent and interactive roles, and explored gender differences, breaking through the simplified treatment of a single dimension of the dormitory atmosphere and the lack of gender heterogeneity in previous studies (Braim et al., 2023; Araujo and Murray, 2021). However, there were still some limitations in our study. First, the analysis was based on cross-sectional data, making it difficult to clarify the causal relationship between dormitory atmosphere and academic achievement. Additionally, the Cronbach’s alpha coefficient for the questionnaire was 0.327. This may be attributed to the fact that Cronbach’s alpha is calculated based on the unidimensional assumption. When applied to scales measuring multiple independent factors, the resulting coefficient fails to accurately reflect the scale’s reliability and may be misleading (Raykov et al., 2024; Barbaranelli et al., 2015). For example, Barbaranelli et al. demonstrated through empirical cases involving The Self-Care of Health Failure (SCHFI) that multiple researchers reported poor alpha coefficients for SCHFI (Barbaranelli et al., 2015; Vellone et al., 2014; Kato et al., 2013; Yu et al., 2011). Meanwhile, the limited number of our questionnaire questions may also lead to this result Furthermore, we adopted the method of convenience sampling when collecting the questionnaires, the “Undergraduate dormitory atmosphere questionnaire” was only distributed in ZCMU, it might have caused selection bias. Future studies should be expanded to include more schools. In addition, dormitory atmosphere indicators rely on students’ subjective reports, answers may be influenced by personal emotions, memory biases, or social expectations (e.g., hiding negative attitudes), reducing objectivity. Other validated methods of assessing dormitory atmosphere should be considered in future studies. Finally, for the above reasons, including the small sample size, the results of our study may be slightly different from those of previous studies. In the future, a longitudinal study with a large sample and objective indicators is worth looking forward to.

## 5 Conclusion

Our study showed that dormitory atmosphere was closely related to students’ AP, especially the hygiene of the dormitory and the interpersonal atmosphere of the dormitory, and emphasized its unique importance among women. Therefore, it is vital to pay attention to the shaping of the atmosphere of college students’ dormitories, which will be of great significance to their future development.

## Data Availability

The original contributions presented in the study are included in the article/Supplementary material, further inquiries can be directed to the corresponding authors.
