# Supplementary material for: The impact of dormitory atmosphere on academic performance in medical university: a cross-sectional study
Source: Front Psychol. 2025 Oct 27;16:1677658. doi: 10.3389/fpsyg.2025.1677658 (PMC12597909; doi:10.3389/fpsyg.2025.1677658)
Supplement: Supplementary file 1 [file Table_1.docx]

**Table S1. Collinearity analysis for all baseline and other controlled variables**

| Model | Nonnormalized coefficient | | Standardization coefficient | t | sig | Collinear statistics | |
| --- | --- | --- | --- | --- | --- | --- | --- |
|  | Beta | SE | Beta |  |  | Tolerance | VIF |
| Sex | -0.157 | 0.048 | -0.162 | -3.249 | 0.001 | 0.585 | 1.709 |
| Campus location | -0.241 | 0.055 | -0.182 | -4.404 | 0.000 | 0.855 | 1.170 |
| Student leadership experience | -0.04 | 0.042 | -0.039 | -0.966 | 0.334 | 0.885 | 1.130 |
| Honors | -0.179 | 0.041 | -0.186 | -4.412 | 0.000 | 0.825 | 1.213 |
| Maternal education | 0.026 | 0.031 | 0.046 | 0.847 | 0.397 | 0.500 | 2.000 |
| Paternal education | -0.008 | 0.031 | -0.015 | -0.273 | 0.785 | 0.480 | 2.083 |
| Monthly expense | -0.037 | 0.078 | -0.019 | -0.480 | 0.632 | 0.945 | 1.058 |
| Learning atmosphere in the family | -0.035 | 0.033 | -0.045 | -1.072 | 0.284 | 0.819 | 1.222 |
| Early to bed | 0.032 | 0.031 | 0.041 | 1.023 | 0.307 | 0.909 | 1.100 |
| Early to rise | -0.031 | 0.045 | -0.029 | -0.695 | 0.487 | 0.866 | 1.155 |
| Study time | -0.111 | 0.057 | -0.077 | -1.956 | 0.051 | 0.933 | 1.072 |
| Dormitory hygiene | -0.120 | 0.043 | -0.125 | -2.781 | 0.006 | 0.719 | 1.391 |
| Dormitory academic atmosphere | -0.025 | 0.039 | -0.026 | -0.65 | 0.516 | 0.884 | 1.131 |
| Dormitory interpersonal atmosphere | 0.088 | 0.037 | 0.092 | 2.389 | 0.017 | 0.980 | 1.021 |

Notes:

SE: Standard Error; t, t-statistic; sig, Significance; VIF, Variance Inflation Factor;
