# Supplementary material for: The impact of dormitory atmosphere on academic performance in medical university: a cross-sectional study
Source: Front Psychol. 2025 Oct 27;16:1677658. doi: 10.3389/fpsyg.2025.1677658 (PMC12597909; doi:10.3389/fpsyg.2025.1677658)
Supplement: Supplementary file 2 [file Table_2.docx]

**Table S2. Undergraduate dormitory atmosphere questionnaire**

| Question | Score | Scoring criteria |
| --- | --- | --- |
| **Demographic Characteristics Questions** | / | / |
| **Socio-demographic characteristics** | / | / |
| Q1(The question of sex) | / | Male  Female |
| Q2(The question of dormitory number) | / | Enter your assigned dorm code: _____ |
| Q3(The question of your mother's highest educational qualification) | / | High school (including general high school and secondary vocational education)/Associate degree  Bachelor’s degree  Master’s degree or above |
| Q4(The question of your father's highest educational qualification) | / | High school (including general high school and secondary vocational education)/Associate degree  Bachelor’s degree  Master’s degree or above |
| Q5(The question of approximate monthly expenditure) | / | $138  $138-$688  > $688 |
| Q6(The question of estimated total books in household) | / | 0-80 books  80-400 books  Over 400 books |
| **Personal ability and habit** | / | / |
| Q7(The question of student leadership experience) | / | Yes  No |
| Q8(The question of academic honors received) | / | Yes  No |
| Q9(The question of frequency of going to bed before 23:30) | / | Seldom  Often  Daily |
| Q10(The question of frequency of waking up before 8:00) | / | Seldom  Often  Daily |
| Q11(The question of the ratio between academic study time and extracurricular activities) | / | 0%  1%-60%  >60% |
| **Dormitory hygiene**  Q12(The question of dormitory cleaning frequency) | 1-10  1-4 | /  Daily = 4 points  Weekly = 3 points  Occasionally = 2 points  Never = 1 points |
| Q13(The question of dormitory hygiene scoring) | 1-3 | Always D = 1 points  Always A = 3 points  Most B or C = 2 points |
| Q14(The question of dormitory hygiene maintenance behaviors)  Behavioral Domains:  1.Routine surface sanitation (e.g., sweeping floors, mopping)  2.Environmental enhancement (e.g., decorative arrangements, plant maintenance)  3.Waste disposal management (e.g., timely removal of filled trash receptacles)  4.Personal space management (e.g., systematic arrangement of clothing and desk items) | 1-4 | Never (0 instances) = 1 points  Infrequently (1-2 instances monthly) = 2 points  Regularly (1-2 instances weekly) = 3 points  Consistently (>2 instances weekly) = 4 points |
| **Dormitory academic atmosphere**  Q15(The question of learning resource sharing frequency) | 1-20  1-4 | /  No sharing event = 1 points  Sharing event in recent week = 4 points  Sharing event in recent month = 3 points  Sharing event in this semester = 2 points |
| Q16(The question of collaborative learning or academic discussing frequency) | 1-5 | Never (No such behavior) = 1 points  Daily (At least once per day) = 5 points  Weekly (1-3 instances per week) = 4 points  Monthly (1-3 instances per month) = 3 points  Per Semester (1-3 instances per semester) = 2 points |
| Q17(The question of your behavioral feedback to roommate success) | 1-4 | be jealous or even belittling = 1 points  not take it seriously = 2 points  envy him or her but not follow him or her as a learning example = 3 points  actively learn from him or her = 4 points |
| Q18(The question of your study hour changes following roommates' high grades) | 1-4 | Virtually negligible impact (with an increase of nearly 0 minutes per day in study time) = 1 points  Slight augmentation (with an increase of approximately 30 - 60 minutes per day in study time)  Moderate escalation (with an increase of roughly 60 - 120 minutes per day in study time) = 3 points  Substantial upsurge (with an increase of 120 minutes or more per day in study time) = 4 points |
| Q19(The question of your leisure adjustment probability during peers' sustained studying) | 1-3 | Low propensity (Maintain leisure activities) = 1 points  Context-dependent adjustment (Modulated by environmental/task factors) = 2 points  High propensity (Actively switch to academic behavior) = 3 points |
| **Dormitory interpersonal atmosphere**  Q20(The question of frequency of interpersonal tension among roommates) | 1-20  1-5 | /  Never (No observable conflicts) = 5 points  Infrequently (1-2 conflict episodes per semester)  Periodically (1-3 conflict episodes monthly) = 3 points  Frequently (1-2 conflict episodes weekly) = 2 points  Persistently (>2 conflict episodes weekly) = 1 points |
| Q21(The question of your general responses to roommate problems) | 1-3 | Ignore it = 1 points  Care a little = 2 points  Help actively = 3 points |
| Q22(The question of frequency of Emotional Connection Among Roommates) | 1-4 | 0-1 day = 1 points  2-3 days = 2 points  3-5 days = 3 points  5-7 days = 4 points |
| Q23(The question of dormitory group activity frequency) | 1-4 | Never = 1 points  Rarely (1-3 times) = 2 points  Occasionally (4-6 times) = 3 points  Frequently (7 times or more) = 4 points |
| Q24(The question of self-regulation when affecting dormitory peers) | 1-4 | Keep doing it = 1 points  Stop after a short while = 2 points  Decide based on the situation = 3 points  Stop immediately = 4 points |

Notes: KMO = 0.737, Cronbach's alpha = 0.327
