# Supplementary material for: The impact of dormitory atmosphere on academic performance in medical university: a cross-sectional study
Source: Front Psychol. 2025 Oct 27;16:1677658. doi: 10.3389/fpsyg.2025.1677658 (PMC12597909; doi:10.3389/fpsyg.2025.1677658)
Supplement: Supplementary file 3 [file Table_3.docx]

**Table S3. Factor structure, internal consistency (Cronbach’s alpha) for factors, corrected item-total correlation, and descriptive imputed data for the final questionnaire (N = 96)**

| Factors and questions | Factor-loading | Corrected item-total correlation | Mean (SD) |
| --- | --- | --- | --- |
| Factor 1: Dormitory hygiene score (alpha = 0.356, explains 44.67% of variance) |  |  |  |
| Q12The question of dormitory cleaning frequency | 0.605 | 0.175 | 2.36 |
| Q13The question of dormitory hygiene scoring | 0.750 | 0.269 | 3.22 |
| Q14The question of dormitory hygiene maintenance behaviors | 0.641 | 0.213 | 2.09 |
| Factor 2: Dormitory academic atmosphere score (alpha = 0.234, explains 55.78% of variance) |  |  |  |
| Q15The question of learning resource sharing frequency | 0.056 | 0.014 | 3.05 |
| Q16The question of collaborative learning or academic discussing frequency | 0.779 | 0.127 | 3.38 |
| Q17The question of your behavioral feedback to roommate success | 0.744 | 0.125 | 2.13 |
| Q18The question of your study hour changes following roommates' high grades | 0.630 | 0.233 | 1.77 |
| Q19The question of your leisure adjustment probability during peers' sustained studying | 0.713 | 0.089 | 1.43 |
| Factor 3: Dormitory interpersonal atmosphere score (alpha = 0.094, explains 68.00% of variance) |  |  |  |
| Q20The question of frequency of interpersonal tension among roommates | 0.748 | 0.133 | 2.28 |
| Q21The question of your general responses to roommate problems | 0.644 | 0.366 | 2.72 |
| Q22The question of frequency of Emotional Connection Among Roommates | 0.592 | 0.418 | 2.73 |
| Q23The question of dormitory group activity frequency | 0.767 | 0.135 | 3.43 |
| Q24The question of self-regulation when affecting dormitory peers | -0.723 | 0.453 | 1.98 |
